# Increased hepatocellular carcinoma risk in chronic hepatitis B patients with persistently elevated serum total bile acid: a retrospective cohort study

**DOI:** 10.1038/srep38180

**Published:** 2016-12-01

**Authors:** Haoliang Wang, Xiaoyun Shang, Xing Wan, Xiaomei Xiang, Qing Mao, Guohong Deng, Yuzhang Wu

**Affiliations:** 1 Institute of Immunology, Third Military Medical University, Chongqing 400038, China; 2 Department of Infectious Diseases, Southwest Hospital, Third Military Medical University, Chongqing 400038, China

## Abstract

To investigate the association between long-term changes of serum total bile acid and hepatocellular carcinoma in chronic hepatitis B patients, we did a retrospective cohort study of 2262 chronic hepatitis B patients with regular antiviral treatment using data from the Hepatitis Biobank at Southwest Hospital Program from 2004 to 2014. Patients in the study were classified into 3 groups according to persistence of elevated serum total bile acid during follow-up: none-low, medium, and high persistence of elevated serum total bile acid. The association between persistence of elevated serum total bile acid and hepatocellular carcinoma was estimated using Cox proportional hazard models and Kaplan-Meier analysis including information about patients’ demographic and clinical characteristics. There were 62 hepatocellular carcinoma cases during a total follow-up of 14756.5 person-years in the retrospective study. Compared to patients with none-low persistence of elevated total bile acid, the multivariate adjusted hazard ratios (95% confidence interval) were 2.37 (1.16–4.84), and 2.57 (1.28–5.16) for patients with medium, and high persistence of elevated total bile acid. Our findings identified persistence of elevated serum total bile acid as an independent risk factor of hepatocellular carcinoma in chronic hepatitis B patients.

Chronic hepatitis B virus (HBV) infection accounts for about 50% of the global hepatocellular carcinoma (HCC) cases^1^. Antiviral treatment has been reported to reduce but not eliminate the risk of HCC in chronic hepatitis B (CHB) patients^2^,^3^,^4^,^5^,^6^. Some risk factors unamenable to antiviral treatment might contribute to HCC development in CHB patients^7^.

Bile acid has been associated with hepatocellular carcinoma (HCC) in human and mice with bile acid receptor deficiency^8^,^9^,^10^, and in mice with altered bile acid metabolism^11^. Elevated serum bile acid has been observed in CHB patients^12^,^13^, while there is currently no epidemiological study to evaluate the independent contribution of elevated serum bile acid to HCC in CHB patient population. A small-sample prospective study shows that HBV-related HCC patients usually had long-term elevated serum bile acid before HCC development^14^. This study provides important information but does not provided enough evidences to support the persistence of elevated serum bile acid as an independent driving factor of HCC in CHB patients, as the authors simply adjusted for demographic factors (age and sex) in the analyses^14^.

Using data from the Hepatitis Biobank at Southwest Hospital Program by the Department of Infectious Diseases at Southwest Hospital, we conducted a retrospective study to evaluate the association between persistence of elevated serum total bile acid (TBA) and HCC in CHB patients. Commonly recognized HCC risk factors, both demographic characteristics (age and sex) and clinical characteristics (liver damage, HBV virus replication, and liver cirrhosis), were included in the analyses as covariates^15^,^16^,^17^,^18^.

## Methods

### Study design and participants

The Hepatitis Biobank at Southwest Hospital Program by the Department of Infectious Diseases at Southwest Hospital covers all the records of patients visiting the Department of Infectious Diseases at Southwest Hospital since 2001. The records include information on the patients’ demographic characteristics, blood test, imaging test, histology test, and treatments. As serum HBV DNA records before 2004 are unavailable in the database, we checked records of the program from 2004 onwards. The study cohort included patients who were positive for serum hepatitis B surface antigen (HBsAg), had a follow-up for at least 4 years, and had serum TBA records in most (more than 90%) of the calendar years of their follow-up. Patients were excluded if they were positive for hepatitis C virus (HCV)/hepatitis D virus (HDV)/human immunodeficiency virus (HIV) infection, if they were diagnosed as autoimmune hepatitis, if they were diagnosed as cancers other than HCC, if they had received liver transplantation/resection surgery before HCC development, if they did not have HBV DNA or hepatitis B e antigen (HBeAg) records during the first year of follow-up, or if they did not have imaging or histology test records. Patients diagnosed as HCC during their first year of follow-up were also excluded to improve the temporal causal relationship evaluation.

Serum HBV DNA and HBeAg as indicators of HBV virus replication, serum alanine aminotransferase (ALT) as indicator of liver damage, liver cirrhosis, age and sex were commonly recognized as HCC risk factors in CHB patients and were chosen as covariates along with serum TBA in this study.

### Verification of HCC

Of the total 62 HCC cases diagnosed during follow-up: 26 cases were verified with a biopsy examination and at least 1 imaging technique (abdominal ultrasonography, computed tomography, or magnetic resonance imaging), 29 cases were verified with at least 2 imaging techniques, and the remaining 7 cases were verified with 1 imaging technique (6 by computed tomography and 1 by ultrasonography) and a serum α–fetoprotein level ≧400 ng/mL.

### Procedures

The scheduled end of follow-up was set at December 31, 2014. The follow-up of each participant began at his first visit to the hospital and ended at HCC diagnosis or censoring. Patients were censored at loss to follow-up (>365 days since the last visit), or at December 31, 2014, whichever came first.

Liver cirrhosis was verified by patients’ image or biopsy test records. We categorized exposure to cirrhosis based on diagnosis of cirrhosis during follow-up, as image or biopsy test data were not available for a large part (1593, 70.4%) of our patients in their first year of follow-up. Of the total 1082 cirrhosis cases diagnosed during follow-up: 901 cases were verified with 1 imaging technique alone, 173 cases were verified with at least two imaging techniques, and 8 cases were verified with biopsy and at least 1 imaging technique. Development of ascites was used as an indicator of the severity of cirrhosis. A total of 145 cirrhotic patients developed ascites during follow-up as revealed by abdominal imaging. Patients’ last aspartate aminotransferase to platelet ratio index (APRI) score in the last year of follow-up was also used to evaluate the liver fibrosis at the end of follow-up^19^. A total of 245 patients without APRI score in the last year of follow-up were excluded from analyses concerning this covariate.

To precisely characterize the long-term changes of serum TBA during follow-up, we divided participants’ follow-up time into separate intervals by 180 days starting from their first visit, as patients in our study cohort usually paid a visit to the hospital at an interval of 3–6 months. The exposure threshold of serum TBA was set at ≧10 μmol/L. The values of serum TBA test results in each 180-day interval were used to evaluate the exposure levels in that 180-day interval. For the 180-day interval without test results, we replaced the missing value with the value from the closest previous visit. The persistence of elevated serum TBA during follow-up was stratified into 3 levels: “none-low persistence of elevated serum TBA” for patients who had elevated serum TBA (≧10 μmol/L) in <1/3 of their total 180-day intervals, “medium persistence of elevated serum TBA” for patients who had elevated serum TBA in 1/3–2/3 of their total 180-day intervals, and “high persistence of elevated serum TBA” for patients who had elevated serum TBA in ≧2/3 of their total 180-day intervals. The persistence of elevated serum ALT and HBV DNA during follow-up was stratified similarly. For serum HBV DNA, the exposure threshold was set at ≧10000 copies/mL according to a previous report^17^. For serum ALT. the exposure threshold was set at ≧45 IU/L. Of the total 31103 180-day intervals created, 25446 (81.8%) 180-day intervals contain serum ALT test results, 25441 (81.8%) 180-day intervals contain serum TBA test results, and 25679 (82.6%) 180-day intervals contain serum HBV DNA test results.

### Statistical analyses

We assessed the characteristics of retrospective cohort by patient groups with different long-term TBA change patterns using κ^2^ test (for categorical data) and Mann-Whitney U test (for continuous data). Cumulative incidence of HCC by patient groups with different long-term TBA change patterns was assessed with Kaplan-Meier analyses, and we tested for crude differences among the groups using a log-rank test. Cox proportional hazard models were used to calculate unadjusted and adjusted hazard ratios and 95% confidence intervals of HCC. STATA software version 13.0 was used for statistical analyses. Statistical significance was set at P < 0.05 and all tests were 2-tailed.

### Human subjects review

The study was approved by the ethics committee of Southwest Hospital (Chongqing, China) and conducted in accordance with The Declaration of Helsinki Principles. Informed consent was obtained from all subjects.

## Results

### Characteristic of retrospective cohort and crude hazard ratios of HCC

**Figure 1:**
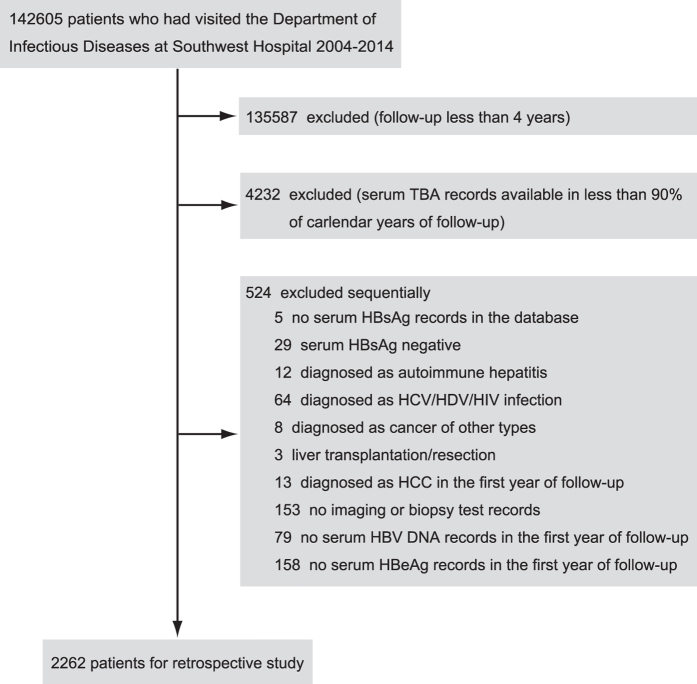
Retrospective study cohort.

There were 142605 patients who have paid at least 1 visit to the Department of Infectious Diseases at Southwest Hospital from 2004 to 2014. A total of 2262 patients met the inclusion criteria and constituted the final retrospective cohort (Fig. 1).

**Figure 2:**
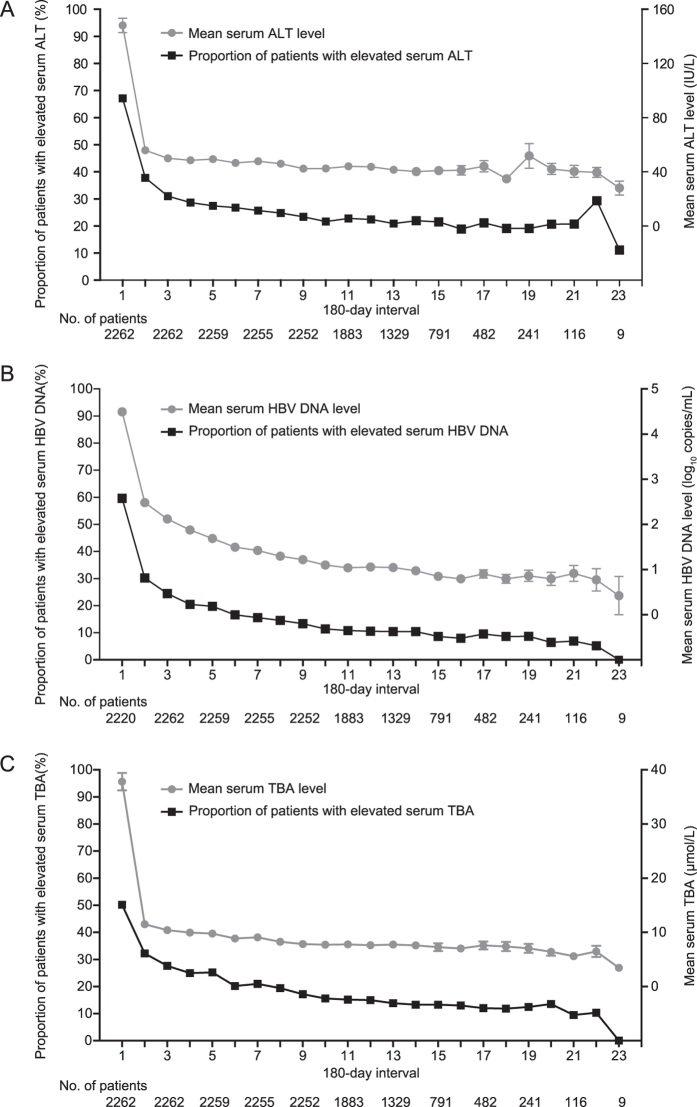
Long-term change of serum ALT, HBV DNA, and TBA. (**A**) Long-term change of serum ALT. Filled rectangles indicate the proportion of patients with elevated serum ALT in each 180-day interval. Filled circles indicate the mean of serum ALT in each 180-day interval and horizontal bars depict standard error. (**B**) Long-term change of serum HBV DNA. Filled rectangles indicate the proportion of patients with elevated serum HBV DNA in each 180-day interval. Filled circles indicate the mean of serum HBV DNA (log transformed) in each 180-day interval and horizontal bars depict standard error. For HBV DNA below the detection limit of 50 copies/mL, HBV DNA level was set to 1 copies/mL before log transforming. A total of 42 patients have no HBV DNA records in the first 180-day interval. (**C**) Long-term change of serum TBA. Filled rectangles indicate the proportion of patients with elevated serum TBA in each 180-day interval. Filled circles indicate the mean of serum TBA in each 180-day interval and horizontal bars depict standard error.

According to the records of drug prescription in the database, most of patients in the cohort had regular antiviral treatment with interferon and/or nucleoside analogue during follow-up. Of the total 2262 patients: only 1 patient had not received any antiviral treatment, 1 patient had received antiviral treatment with interferon alone, and the rest 2260 patients had received antiviral treatment with both interferon and nucleoside analogue. For the total 2261 patients with antiviral treatment, 2259 patients had antiviral treatment in more than 50% of the calendar years of their follow-up. Figure 2 shows the changes in the serum ALT, HBV DNA and TBA levels during follow-up. The serum ALT, HBV DNA, and TBA level decreased progressively with the most marked decline during the initial follow-up possibly due to antiviral treatment (Fig. 2).

The characteristics of our retrospective cohort and their association with HCC by univariate analysis are shown in Table 1. A total of 62 HCC cases were diagnosed during a total follow-up of 14756.5 person-years. Medium, and high persistence of elevated serum TBA was observed in 267 (11.8%), and 222 (9.8%) patients. Compared with patients with none-low persistence of elevated serum TBA, the univariate hazard ratios were 3.70 (95% confidence interval, 1.89–7.22; P < 0.001), and 8.60 (95% confidence interval, 4.91–15.06; P < 0.001) for patients with medium, and high persistence of elevated serum TBA. Medium, and high persistence of elevated serum ALT was observed in 589 (26.0%), and 238 (10.5%) patients. Compared with patients with none-low persistence of elevated serum ALT, the univariate hazard ratios were 1.78 (95% confidence interval, 1.01–3.14; P = 0.04), and 2.93 (95% confidence interval, 1.52–5.66; P = 0.001) for patients with medium, and high persistence of elevated serum ALT. Medium, and high persistence of elevated serum HBV DNA was observed in 316 (14.0%), and 139 (6.2%) patients. Compared with patients with none-low persistence of elevated serum HBV DNA, the univariate hazard ratios were 1.25 (95% confidence interval, 0.63–2.47; P = 0.52), and 1.16 (95% confidence interval, 0.42–3.23; P = 0.77) for patients with medium, and high persistence of elevated serum HBV DNA. A total of 1082 (47.8%) patients were diagnosed as liver cirrhosis, and 145 of them had developed ascites during the follow-up. Compared with non-cirrhotic patients, the univariate hazard ratios were 31.26 (95% confidence interval, 7.59–128.82; P < 0.001), and 62.38 (95% confidence interval, 14.17–274.51; P < 0.001) for cirrhotic patients without ascites, and cirrhotic patients with ascites. For 2017 patients who had APRI records in their last year of follow-up, 732 (36.3%) patients had the last APRI score 0.5–1.5, and 174 (8.6%) patients had the last APRI score >1.5. Compared with patients with the last APRI score <0.5, the univariate hazard ratios were 3.47 (95% confidence interval, 1.76–6.85; P < 0.001), and 12.10 (95% confidence interval, 5.91–24.77; P < 0.001) for patients with the last APRI score 0.5–1.5, and patients with the last APRI score >1.5. About half of patients (1107, 48.9%) were HBeAg serum positive at baseline. Compared with patients with negative serum HBeAg at baseline, the univariate hazard ratio was 0.44 (95% confidence interval, 0.25–0.76; P = 0.003) for patients with positive serum HBeAg at baseline.

### Cumulative incidence of HCC by persistence of elevated serum TBA

**Figure 3:**
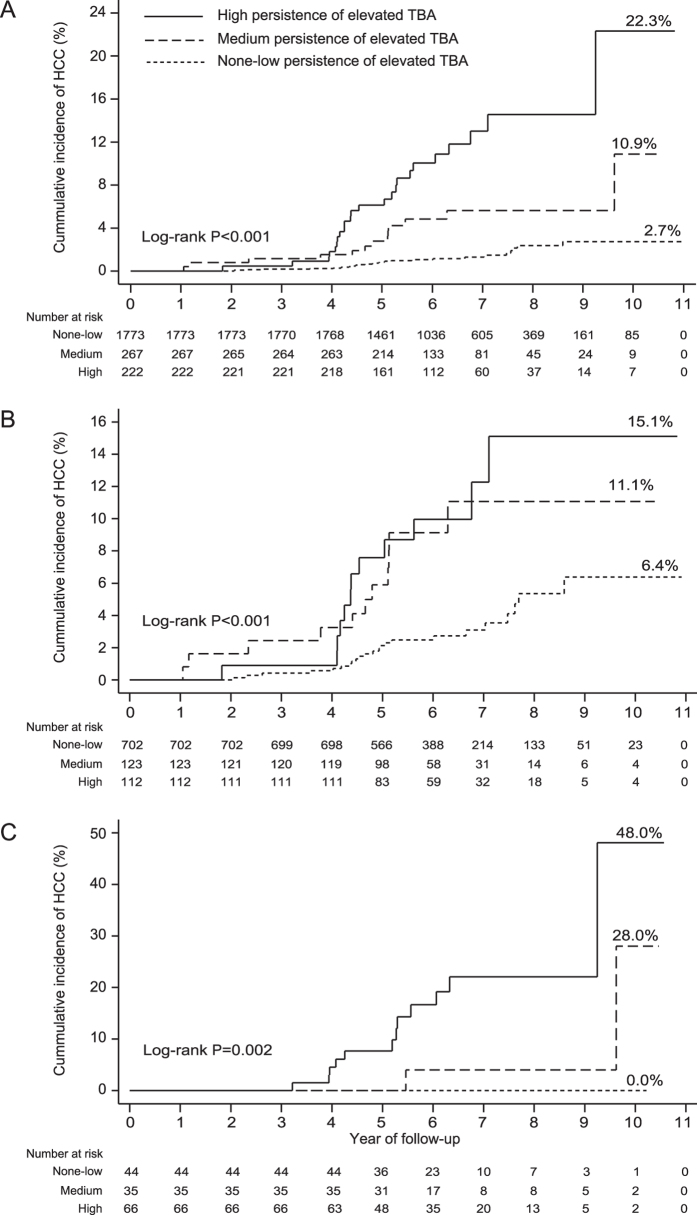
Cumulative incidence of HCC, by persistence of elevated serum TBA. (**A**) The whole cohort. (**B**) The sub-cohort of 937 cirrhotic patients without ascites during follow-up. (**C**) The sub-cohort of 145 cirrhotic patients with ascites during follow-up.

For the whole cohort, the cumulative probability of HCC at the end of follow-up was 2.7%, 10.9%, and 22.3% for patient with none-low, medium, and high persistence of elevated serum TBA, respectively (Log-rank P < 0.001) (Fig. 3A). For the sub-cohort of 937 cirrhotic patients without ascites, the cumulative probability of HCC at the end of follow-up was 6.4%, 11.1%, and 15.1% for patient with none-low, medium, and high persistence of elevated serum TBA, respectively (Log-rank P < 0.001) (Fig. 3B). For the sub-cohort of 145 cirrhotic patients with ascites, the cumulative probability of HCC at the end of follow-up was 0.0%, 28.0%, and 48.0% for patient with none-low, medium, and high persistence of elevated serum TBA, respectively (Log-rank P = 0.002) (Fig. 3C).

### Characteristics of retrospective cohort by persistence of elevated serum TBA

The characteristics of patients groups with different persistence of elevated serum TBA are shown in Table 2. Compared with patients with none-low persistence of elevated serum TBA, patients with medium or high persistence of elevated serum TBA were more likely to have shorter length of follow-up, to have older age at entry, to be diagnosed with cirrhosis and ascites during follow-up, to have higher APRI scores at the end of follow-up, and to have medium or high persistence of elevated serum ALT (P < 0.05) (Table 2).

### Multivariate-adjusted hazard ratios of HCC

Multivariate analyses adjusting for all the covariates listed in Table 1 are shown in Table 3. A total of 245 patients without APRI score in the last year of follow-up were excluded from multivariate analyses. Compared with patients with none-low persistence of elevated serum TBA, the multivariate adjusted hazard ratios were 2.37 (95% confidence interval, 1.16–4.84; P = 0.018), and 2.57 (95% confidence interval, 1.28–5.16; P = 0.008) for patients with medium, and high persistence of elevated serum TBA (Table 3). Compared with non-cirrhotic patients, the multivariate hazard ratios were 17.01 (95% confidence interval, 3.96–73.15; P < 0.001), and 15.42 (95% confidence interval, 3.20–74.32; P = 0.001) for cirrhotic patients without ascites, and cirrhotic patients with ascites (Table 3). Compared with patients with the last APRI score <0.5, the multivariate hazard ratios were 1.60 (95% confidence interval, 0.78–3.27; P = 0.20), and 2.67 (95% confidence interval, 1.12–6.38; P = 0.027) for patients with the last APRI score 0.5–1.5, and patients with the last APRI score >1.5 (Table 3). Compared with patients with none-low persistence of elevated serum HBV DNA, the multivariate hazard ratios were 1.62 (95% confidence interval, 0.78–3.35; P = 0.19), and 3.14 (95% confidence interval, 1.02–9.64; P = 0.045) for patients with medium, and high persistence of elevated serum HBV DNA (Table 3). Age at entry was significantly associated with HCC (P = 0.027) (Table 3). Sex, persistence of elevated serum ALT, and baseline HBeAg were not significantly associated with HCC in the multivariate analyses (P > 0.05) (Table 3).

## Discussion

Our retrospective study identified persistently elevated serum TBA as a major independent risk factor for HCC development in CHB patients receiving regular antiviral treatment. A total of 37 (59.7%) HCC cases in our cohort were contributed by the patient populations with medium or high persistence of elevated serum TBA.

Previous epidemiological studies identified the persistence of elevated serum ALT/HBV DNA as major risk factors of HCC in CHB patient population without antiviral treatment^18^. Our multivariate analyses also found significant association between persistence of elevated serum HBV DNA and HCC. However, in our multivariate analyses, only patients with high persistence of elevated serum HBV DNA showed significantly increased HCC risk compared with patients with none-low persistence of elevated serum HBV DNA, and only 4 (6.9%) HCC cases were contributed by this patient population (Table 3). The decreased HCC risk burden by persistence of elevated serum HBV DNA in our cohort might be explained by the regular antiviral treatment in our patients. Unlike previous study^18^, although our univariate analyses showed significant association between persistence of elevated serum ALT and HCC (Table 1), our multivariate analyses did not show significant association between persistence of elevated serum ALT and HCC (Table 3). This discrepancy might result from the different ways of adjusting for cirrhosis in the analyses. Previous study adjusts for cirrhosis by excluding patients with cirrhosis at enrollment^18^, which would inevitably lead to an underestimation of the effect of cirrhosis on HCC development as most of HBV-related HCC cases (70–90%) occur in patients with cirrhosis^1^. Our study not only adjusts for diagnosis of cirrhosis during the follow-up but also adjusts for the severity of cirrhosis through ascites and APRI score at the end of follow-up. There is significant association between persistence of elevated serum ALT and APRI score at the end of follow-up in our cohort (P < 0.001). The loss of significant association between persistence of elevated serum ALT and HCC in the multivariate analyses indicates that persistence of elevated serum ALT is not an independent risk factor of HCC when the severity of cirrhosis is taken into consideration.

Bile acid, as product of cholesterol metabolism, is traditionally recognized as detergents for dietary lipid digestion and absorption, while accumulative evidences have identified its role as hormone with distinct receptors in multiple tissues^20^. Previous studies from mice model reveals a pro-inflammatory role of bile acid^21^,^22^, which is thought to cause HCC development by promoting genomic modification of hepatocytes^11^,^23^. Except for hepatocyte damage, elevated serum bile acid in CHB patients could also be caused by disrupted bile acid metabolism: Binding of HBV virus to bile acid receptor Na^+^-taurocholate cotransporting polypeptide (NTCP) could promote hepatic bile acid synthesis^24^; Bile acid metabolism could also be modulated by gut microbiota in cirrhotic patients^25^.

Our results should be interpreted within the study’s limitations. Firstly, for lack of image test results for a large part of our patients in the initial follow-up, our study adjusts for cirrhosis based on diagnosis of cirrhosis during follow-up. This shortcoming might overestimate the effect of cirrhosis on HCC because some cirrhotic patients might be free of cirrhosis at entry. Of the 60 HCC cases with cirrhosis in our cohort, 37 (61.7%) cases were diagnosed as cirrhosis at least 1 year after the start of follow-up. Secondly, as persistence of elevated serum TBA was closely associated with diagnosis of cirrhosis during follow-up in our cohort, it is reasonable to suspect that the increased HCC risk in patients with medium or high persistence of elevated serum TBA might simply be a result of advanced cirrhosis in those patients. To address this issue, we included ascites and APRI score into our analyses. Development of ascites is a clinical feature of advanced cirrhosis. The APRI scoring system is less applicable in CHB patients without antiviral treatment due to severe hepatic inflammation^26^. As the hepatic inflammation decreased progressively in our cohort due to regular antiviral treatment, we used patients’ last APRI score to evaluate liver fibrosis at the end of follow-up. Inclusion of those two factors in the analyses supported the role of persistence of elevated serum TBA as an independent driving factor for HCC. Also worthy of notice, the close association between persistence of elevated serum TBA and severity of cirrhosis in our data do not imply a causal relationship between those two factors, as there is no reliable information about the severity of cirrhosis during initial follow-up of our cohort. Thirdly, due to lack of data, our analyses did not adjust for HBV genotype and HBV mutations. Both HBV genotype and HBV mutation in the precore/enhancer II region and the preS region have been reported to be independent HCC risk factors in CHB patients^16^,^27^,^28^,^29^,^30^,^31^. It is not clear whether persistence of elevated serum TBA is associated with specific HBV genotypes or specific HBV variants in our cohort.

Our findings identified persistence of elevated serum TBA as an independent risk factor of HCC in CHB patients with regular antiviral treatments, which could help to improve routine surveillance for HCC in current clinical practice. Accumulative metabolomics data has found elevated serum bile acid as a core metabolomics phenotype in almost all the types of liver diseases^32^. It is worthy to apply our findings on those liver diseases.

## Additional Information

**How to cite this article**: Wang, H. *et al*. Increased hepatocellular carcinoma risk in chronic hepatitis B patients with persistently elevated serum total bile acid: a retrospective cohort study. *Sci. Rep.* **6**, 38180; doi: 10.1038/srep38180 (2016).

**Publisher's note:** Springer Nature remains neutral with regard to jurisdictional claims in published maps and institutional affiliations.

## Figures and Tables

**Table 1:** Demographical and clinical characteristics of retrospective cohort and their association with HCC.

Characteristics	Participants, number (%)	Follow-up, person-years	HCC, number	Hazard ratio (95% confidence interval)	P value
Age at entry, y					
<30	692 (30.6)	4581.8	8	1.00 [Reference]	
30–39	941 (41.6)	6204.1	20	1.84 (0.81–4.18)	0.14
40–49	433 (19.1)	2762.6	18	3.81 (1.66–8.77)	0.002
≧50	196 (8.7)	1208.0	16	7.95 (3.40–18.60)	<0.001
Sex					
Female	552 (24.4)	3541.5	11	1.00 [Reference]	
Male	1710 (75.6)	11215.0	51	1.44 (0.75–2.76)	0.28
Cirrhosis					
Non-cirrhosis	1180 (52.2)	7863.8	2	1.00 [Reference]	
Cirrhosis without ascites	937 (41.4)	5975.3	46	31.26 (7.59–128.82)	<0.001
Cirrhosis with ascites	145 (6.4)	917.4	14	62.38 (14.17–274.51)	<0.001
APRI at the end					
<0.5	1111 (55.1)	7335.9	12	1.00 [Reference]	
0.5–1.5	732 (36.3)	4776.9	27	3.47 (1.76–6.85)	<0.001
>1.5	174 (8.6)	1064.2	20	12.10 (5.91–24.77)	<0.001
Persistence of elevated TBA					
None-low	1773 (78.4)	11690.3	25	1.00 [Reference]	
Medium	267 (11.8)	1686.7	13	3.70 (1.89–7.22)	<0.001
High	222 (9.8)	1379.5	24	8.60 (4.91–15.06)	<0.001
Persistence of elevated ALT					
None-low	1435 (63.4)	9357.4	28	1.00 [Reference]	
Medium	589 (26.0)	3892.3	21	1.78 (1.01–3.14)	0.04
High	238 (10.5)	1506.8	13	2.93 (1.52–5.66)	0.001
Persistence of elevated HBV DNA					
None-low	1807 (79.9)	11856.6	48	1.00 [Reference]	
Medium	316 (14.0)	2020.5	10	1.25 (0.63–2.47)	0.52
High	139 (6.2)	879.4	4	1.16 (0.42–3.23)	0.77
Baseline HBeAg					
Negative	1155 (51.1)	7592.4	44	1.00 [Reference]	
Positive	1107 (48.9)	7164.1	18	0.44 (0.25–0.76)	0.003

**Table 2:** Characteristics of retrospective cohort, by persistence of elevated serum TBA.

Characteristics	None-low persistence of elevated TBA (n = 1773), number (%) or median (interquartile range)	Medium persistence of elevated TBA (n = 267), number (%) or median (interquartile range)	P value, medium versus none-low	High persistence of elevated TBA (n = 222), number (%) or median (interquartile range)	P value, high versus none-low
Loss to follow-up	462 (26.1)	83 (31.1)	0.08	68 (30.6)	0.15
Follow-up, y	6.4 (5.3–7.6)	6.0 (5.1–7.3)	0.001	6.0 (4.9–7.1)	0.001
Age at entry, y					
<30	586 (33.0)	78 (29.2)		28 (12.6)	
30–39	759 (42.8)	94 (35.2)		88 (39.6)	
40–49	294 (16.6)	73 (27.3)	0.001	66 (29.7)	<0.001
≧50	134 (7.6)	22 (8.2)		40 (18.0)	
Sex					
Female	452 (25.5)	55 (20.6)		45 (20.3)	
Male	1321 (74.5)	212 (79.4)	0.08	177 (79.7)	0.09
Cirrhosis					
Non-cirrhosis	1027 (57.9)	109 (40.8)		44 (19.8)	
Cirrhosis without ascites	702 (39.6)	123 (46.1)	<0.001	112 (50.4)	<0.001
Cirrhosis with ascites	44 (2.5)	35 (13.1)		66 (29.7)	
APRI at the end					
<0.5	965 (61.5)	99 (40.9)		47 (22.7)	
0.5–1.5	539 (34.4)	115 (47.5)	<0.001	78 (37.7)	<0.001
>1.5	64 (4.1)	28 (11.6)		82 (39.6)	
Persistence of elevated ALT					
None-low	1166 (65.8)	145 (54.3)		124 (55.9)	
Medium	440 (24.8)	86 (32.2)	0.001	63 (28.4)	0.003
High	167 (9.4)	36 (13.5)		35 (15.8)	
Persistence of elevated HBV DNA					
None-low	1419 (80.0)	208 (77.9)		180 (81.1)	
Medium	242 (13.6)	45 (16.8)	0.32	29 (13.1)	0.93
High	112 (6.3)	14 (5.2)		13 (5.9)	
Baseline HBeAg					
Serum negative	882 (49.8)	140 (52.4)		133 (59.9)	
Serum positive	891 (50.2)	127 (47.6)	0.41	89 (40.1)	0.004

**Table 3:** Multivariate analyses of risk for HCC.

Characteristics	HCC, number (%)	Hazard ratio (95% confidence interval)	P value
Age at entry in 1-year increment		1.03 (1.00–1.06)	0.027
Sex			
Female	11 (18.6)	1.00 [Reference]	
Male	48 (81.4)	1.17 (0.58–2.39)	0.66
Cirrhosis			
Non-cirrhosis	2 (3.4)	1.00 [Reference]	
Cirrhosis without ascites	43 (72.9)	17.01 (3.96–73.15)	<0.001
Cirrhosis with ascites	14 (23.7)	15.42 (3.20–74.32)	0.001
APRI at the end			
<0.5	12 (20.3)	1.00 [Reference]	
0.5–1.5	27 (45.8)	1.60 (0.78–3.27)	0.20
>1.5	20 (33.9)	2.67 (1.12–6.38)	0.027
Persistence of elevated TBA			
None-low	23 (39.0)	1.00 [Reference]	
Medium	13 (22.0)	2.37 (1.16–4.84)	0.018
High	23 (39.0)	2.57 (1.28–5.16)	0.008
Persistence of elevated ALT			
None-low	28 (47.4)	1.00 [Reference]	
Medium	18 (30.5)	1.26 (0.67–2.39)	0.47
High	13 (22.0)	1.82 (0.86–3.90)	0.12
Persistence of elevated HBV DNA			
None-low	45 (76.3)	1.00 [Reference]	
Medium	10 (16.9)	1.62 (0.78–3.35)	0.19
High	4 (6.8)	3.14 (1.02–9.64)	0.045
Baseline HBeAg			
Negative	43 (72.9)	1.00 [Reference]	
Positive	16 (27.1)	0.61 (0.33–1.13)	0.12
